# Particle-size dependent effects in the Balb/c murine model of inhalational melioidosis

**DOI:** 10.3389/fcimb.2012.00101

**Published:** 2012-07-23

**Authors:** Richard J. Thomas, C. Davies, A. Nunez, S. Hibbs, L. Eastaugh, S. Harding, J. Jordan, K. Barnes, P. Oyston, S. Eley

**Affiliations:** ^1^Department of Biomedical Sciences, Defence Science and Technology Laboratory, SalisburyWiltshire, UK; ^2^Veterinary Laboratory Agencies, Weybridge, New HawAddlestone, UK

**Keywords:** *Burkholderia pseudomallei*, aerosol, particle size, melioidosis

## Abstract

Deposition of *Burkholderia pseudomallei* within either the lungs or nasal passages of the Balb/c murine model resulted in different infection kinetics. The infection resulting from the inhalation of *B. pseudomallei* within a 12 μm particle aerosol was prolonged compared to a 1 μm particle aerosol with a mean time-to-death (MTD) of 174.7 ± 14.9 h and 73.8 ± 11.3 h, respectively. Inhalation of *B. pseudomallei* within 1 μm or 12 μm particle aerosols resulted in a median lethal dose (MLD) of 4 and 12 cfu, respectively. The 12 μm particle inhalational infection was characterized by a marked involvement of the nasal mucosa and extension of bacterial colonization and inflammatory lesions from the olfactory epithelium through the olfactory nerves (or tracts) to the olfactory bulb (100%), culminating in abscessation of the brain (33%). Initial involvement of the upper respiratory tract lymphoid tissues (nasal-associated lymphoid tissue (NALT) and cervical lymph nodes) was observed in both the 1 and 12 μm particle inhalational infections (80–85%). Necrotising alveolitis and bronchiolitis were evident in both inhalational infections, however, lung pathology was greater after inhalation of the 1 μm particle aerosol with pronounced involvement of the mediastinal lymph node (50%). Terminal disease was characterized by bacteraemia in both inhalational infections with dissemination to the spleen, liver, kidneys, and thymus. Treatment with co-trimoxazole was more effective than treatment with doxycycline irrespective of the size of the particles inhaled. Doxycycline was more effective against the 12 μm particle inhalational infection as evidenced by increased time to death. However, both treatment regimes exhibited significant relapse when therapy was discontinued with massive enlargement and abscessation of the lungs, spleen, and cervical lymph nodes observed.

## Introduction

*Burkholderia pseudomallei*, the causative agent of melioidosis in humans is endemic to Southeast Asia and northern Australia. A range of potentially fatal presentations can occur ranging from chronic to acute and sub-acute disease with secondary pneumonia due to haematogenous seeding a common manifestation. However, primary pneumonia can also develop through direct inhalation of airborne bacteria (Gilad, 2007; Limmathurotsakul and Peacock, 2011). This can be evidenced by the association of pneumonic infection with heavy rainfalls in Australia and the exposure of soldiers to contaminated dust aerosolized by the action of helicopter rotors in Vietnam (Howe et al., 1971; Currie and Jacups, 2003; Cheng et al., 2006, 2008).

Aerosol particle size affects where micro-organisms deposit within the continuum of the respiratory tract. Furthermore, it has been demonstrated for a number of pathogens in a range of animal infection models that aerosol particle size influences pathogenesis (Druett et al., 1953, 1956a,b; Day and Berendt, 1972; Thomas et al., 2010). The pneumonic inhalational model of melioidosis has been extensively studied in murine models (Jeddeloh et al., 2003; Tan et al., 2008; Titball et al., 2008; Lever et al., 2009). Recently, the first non-human primate model has been published based on the marmoset (Nelson et al., 2011). Evidence for differential pathology resulting from deposition of *B. pseudomallei* within the upper respiratory tract was provided by the study of Owen et al. (2009) where bacteria instilled through the nasal cavity colonized and replicated within the nasal cavity and nasal-associated lymphoid tissue (NALT). Infection of the olfactory epithelium and brain was observed prior to detection of the bacteria within the bloodstream indicating an alternative route of entry into the brain as opposed to haematogenous dissemination.

Melioidosis is difficult to treat due to the intrinsic resistance of the bacterium to antibiotics, partly due to occupation of an intracellular niche and partly due to the possession of a range of resistance mechanisms including efflux pumps, degradative enzymes, and mutational changes in antibiotic targets (Moore et al., 1999; Tribuddharat et al., 2003; Mima and Schweizer, 2010). Abscessation adds complexity as antibiotics experience difficulties reaching minimum inhibitory concentrations for clinically relevant periods of time (Wagner et al., 2006).

This study determined the influence of aerosol particle size on the pathogenesis of inhalational melioidosis in a murine infection model and assessed the efficacy of antimicrobial chemotherapy against the resultant infections.

## Materials and methods

### Animal care

Female Balb/c mice (Charles River, UK) were housed with access to food and water *ad libitum* at ACDP (Advisory Committee on Dangerous Pathogens) biological safety level (BSL)-3. Procedures were performed in accordance with the Scientific Procedures (Animals) Act 1986 and the Codes of Practice for the Housing and Care of Animals Used in Scientific Procedures, 1989. All mice were 6–8 weeks old and weighing 20–25 g upon commencement of aerosol exposures.

### Preparation of *B. pseudomallei* K96243 for aerosol challenge

*B. pseudomallei* was obtained from the Defence Science and Technology Laboratory culture collection (Dstl Porton Down, Salisbury, Wiltshire, UK). Stock cultures were maintained by at −80°C in nutrient broth containing 10% (vol/vol) glycerol. Enumeration was routinely performed on Columbia Blood agar plates at 37°C for 24 h. For aerosol exposures, nutrient broth cultures were shaken at 120 revolutions min^−1^ for 24 h at 37°C. The required dilutions were prepared in nutrient broth in 10 ml volumes immediately prior to challenge.

### Aerosol exposures

Groups of 10 mice were nose-only exposed for a period of 10 min to aerosols generated by the Collison nebuliser or flow-focusing aerosol generator (FFAG) according to the methodology previously described (Thomas et al., 2008, 2009). Aerosol samples from the challenges involving the Collison nebuliser were collected for 1 min into an all-glass impinger (AGI-30; Ace Glass Inc., NJ) containing 10 ml of phosphate buffered saline (PBS) at a flow rate of 12 l min^−1^. The impinger samples were serially diluted and plated onto Columbia blood agar for enumeration. All exposures were performed within a rigid unpolymerized polyvinylchloride (PVCu) half-suit isolator at BSL-3. The mass median aerosol diameters (MMAD) of the particles were 1–3 μm and 12 μm, respectively, for the Collison nebuliser and the FFAG. The mean calculated inhaled dose for small particles was determined using the impinger data and Guyton's formula (Guyton, 1947). The actual mean inhaled dose for small or large particles was derived from physical washing or dissection and homogenization of the nasal passages, trachea, and lungs as described below.

### Deposition and infection kinetics

At specific time-points, five mice were culled by intraperitoneal administration of 0.5 ml sodium pentobarbital (0 h) or halothane intoxication (remaining time-points) and lungs, mediastinal lymph node, Peyer's patches, gastrointestinal lymph nodes (mesenteric, jejunal, colonic), trachea, oesophagus, stomach, intestine, liver, and kidneys aseptically collected. Blood was collected by cardiac puncture after halothane anaesthetization into heparin coated plasma tubes (BD Biosciences, UK). Bacteria deposited in the nasal passages (nasal washings) were collected by inserting a catheter (outer and inner diameter 1.02 and 0.58 mm, respectively; Harvard Apparatus) into a tracheal incision and flushing with 1 ml of PBS containing final concentrations of 1 mM NaOH and 0.1% (v/v) Triton-X100. Tissue samples were homogenized in PBS (Gibco®, Invitrogen Ltd., UK). All samples were enumerated by serial dilution and plating of 100 μ l aliquots in triplicate onto Columbia blood agar plates.

### Determination of median lethal dose (MLD)

Groups of 10 mice were exposed nose-only for 10 min to aerosolized *B. pseudomallei* K96243 at five different 10-fold dilutions generated by either the Collison nebuliser or the FFAG decreasing from the “neat” suspension of 10^9^ cfu ml^−1^. Mice were monitored for 21 d post-exposure for signs of infection. Mice showing signs of infection were culled by cervical dislocation at defined humane end-points. The MLD was calculated by the 50% end-point method (Reed and Muench, 1938), based on the number of bacteria retained in the lungs or nasal passages of animals that received the neat suspension. Immediately after exposure, five animals per dose were sacrificed for necropsy with removal of the lungs, trachea, oesophagus, and stomach. The nasal passages were washed as previously described. The number of bacteria retained in the lungs or nasal passages was demonstrated to decrease by one logarithm with each logarithmic dilution of the spray suspension used in the dilution series.

### Histopathology

The mice were dissected to expose the abdominal and thoracic cavities. The lungs were inflated by instillation of 1.5 mL 10% (v/v) neutral-buffered formalin (NBF) to provide a pressure of 25–30 cm^3^ H_2_O. The whole mouse was fixed in 10% (v/v) NBF for 6 d with replacement with a fresh aliquot after 3 d. After a further 3 d fixation, samples from a range of tissues including lungs (apical, medium, and caudal lobes), lymph nodes (mediastinal, submandibular/cervical, inguinal, and mesenteric nodes), nasal cavity including NALT and turbinates, pharynx, trachea, thymus, thyroid, eyes, brain (including olfactory bulb), oesophagus, stomach, intestine, jejunum containing Peyer's patches, spleen, liver, kidneys, and sternal bone marrow were blocked and embedded in paraffin wax. Serial 4 μm thick sections were cut using a Leica RM2025 microtome (Leica Microsystems Ltd., UK) and dried onto charged slides at 37°C overnight. Sections were stained with haematoxylin and eosin or Gram Twort before examination using a Leica DM4000B microscope. Images were taken using a Leica DFC480 digital camera (Leica Microsystems Ltd., UK). Lesions were scored on the basis of the number of animals displaying specific pathological changes and the severity of these lesions according to the system previously described (Thomas et al., 2009).

### Efficacy of antimicrobial chemotherapy

Groups of 10 mice challenged with either small or large particle aerosols (10 or 100 MLDs) containing *B. pseudomallei* K96243 were administered doxycycline or co-trimoxazole orally for 14 days. Doxycycline oral suspension (Sigma-Aldrich, UK) was administered to mice daily at 1 mg/mouse (50 mg/kg). Co-trimoxazole oral suspension (Septrin®, Glaxo SmithKline Laboratories, UK) was administered to mice twice daily at 4.8 mg/mouse (240 mg/kg). Control groups of 10 infected, non-treated mice were given sterile deionized water orally for 14 days. All mice were observed twice daily for clinical signs of disease for up to 96 days post-infection and mortality recorded. Doses were derived from Food and Drugs Agency Guidance using inversed scaling based on body weight (FDA, 2005).

### Statistical analysis

Data are expressed as the standard error (S.E) around the mean. Tissues containing bacterial loads below the limit of detection (<10 cfu per organ) were designated culture-negative. Two-sample *t*-test was used to test the significant differences in the deposition data. The significant differences in the antibiotic studies was analysed by ANOVAs followed by Tukey's honestly significant difference post-test. *P* values of <0.05 are considered significantly different.

## Results

### *Burkholderia pseudomallei* can produce infection after deposition in the URT

**Figure 1:**
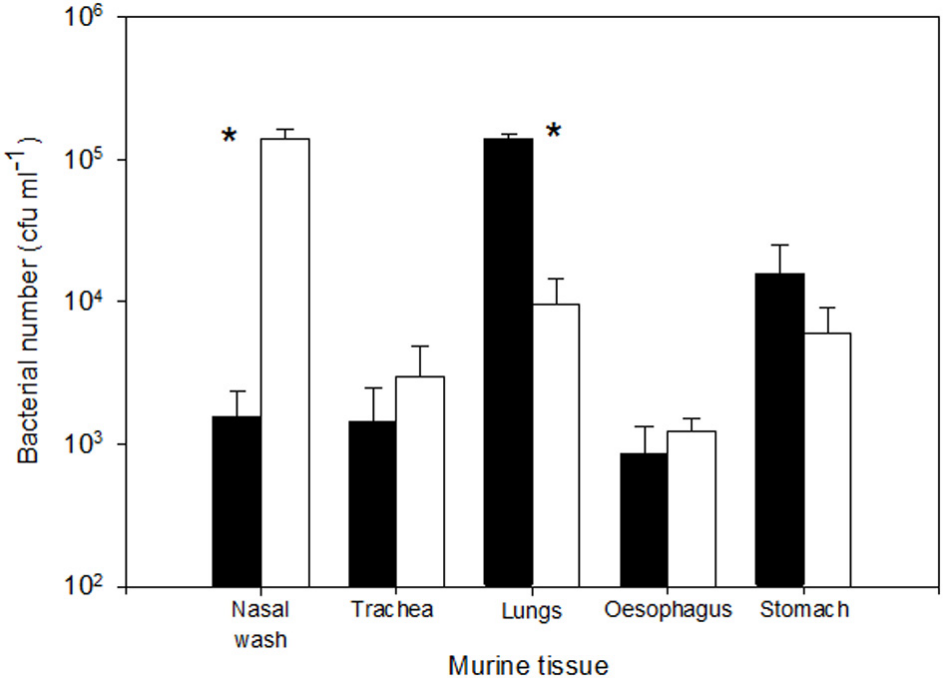
**Initial deposition of *B. pseudomallei* K96243 after exposure to 1 or 12 μm particle aerosols (shaded and open bars respectively); values represent 30 min post-exposure.** Error bars represent the standard errors (*n* = 10). Asterisks represent significant difference between particle sizes.

The regional deposition profiles of *B. pseudomallei* were determined to evaluate where the organisms would predominantly deposit when inhaled within 1 or 12 μm particles (Figure 1). A culture containing 2.95 ± 0.35 × 10^9^ cfu ml^−1^ of *B. pseudomallei* was aerosolized and the retained dose determined. When the 1 μm particle aerosol was generated, impinger samples indicated a retained bacterial dose of 2.79 × 10^5^ cfu. The actual retained dose determined at post-mortem was 1.40 ± 0.09 × 10^5^ cfu in lung tissues and 1.56 ± 0.77 × 10^3^ cfu in the nasal passages. Hence, 87.7% and 1.0% of the total deposited fraction were retained in the lungs and nasal passages, respectively; this was a significant difference (*p* = 0.0002). When a 12 μm particle aerosol was generated, 1.40 ± 0.22 × 10^5^ and 9.50 ± 5.2 × 10^3^ cfu were recovered at post-mortem from the nasal passages and lungs, respectively. This was a significant difference, equivalent to 87.6% and 6.0% of the total retained bacteria being present within the nasal passages and lungs respectively (*p* = 0.0006). The percentage of total deposited bacteria recovered from the stomach was much reduced at 9.9% and 3.8% for mice that inhaled the 1 or 12 μm particles, respectively. The numbers of bacteria present in the trachea and oesophagus was less than 2% of the total retained fraction irrespective of the size of particle inhaled. No significant differences were observed between 1 and 12 μm particle aerosols for the numbers of *B. pseudomallei* deposited in the trachea, oesophagus, and stomach (*p* > 0.09). Ten-fold serial dilutions of the initial challenge culture resulted in approximate 10-fold lower numbers deposited in the respiratory tracts of mice (data not shown).

The MLD of *B. pseudomallei* was determined to investigate the number of bacteria required to generate infection in the URT and LRT of mice challenged with 1 or 12 μm particles Mice with a retained dose of 10^3^ cfu of 1 μm particles displayed a MTD of 52.8 ± 2.57 h, whilst a retained dose of 10^2^ cfu resulted in protracted infection with all of the mice succumbing by 22 d post-infection with a MTD of 146.9 ± 37.5 h post-infection. Mice with retained doses of 1 cfu resulted in 30% survival. When mice received a retained dose of 3 × 10^3^ cfu of *B. pseudomallei* in 12 μm particles all of the animals died with a MTD of 91.9 ± 17.5 h whilst a retained dose of 30 cfu resulted in protracted infection with 90% of the mice succumbing by 21 d post-infection with a MTD of 259.5 ± 33.6 h. Only 20% of mice that received a retained dose of 3 cfu succumbed to infection. The mice that survived until the end of the experiment had cleared the infection as evidenced by bacteriological analysis of lung and spleen tissues. Irrespective of particle size, none of the mice challenged with concentrations predicted to contain less than a single viable bacterium died during the course of the experiment. Calculation of the MLD produced values of 4 and 12 cfu, respectively for *B. pseudomallei* in 1 or 12 μm particles.

### Pathogenesis of inhalational melioidosis differs depending on the site of deposition

**Figure 2:**
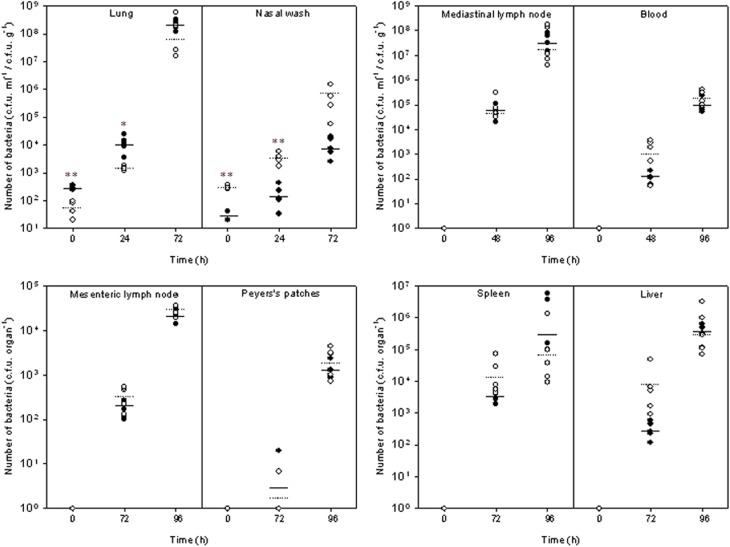
**Bacteriological kinetics within tissues of Balb/c mice exposed to aerosols of different particle sizes containing *Burkholderia pseudomallei* K96243.** •, Small particle (1 μm) infection generated by the Collison nebuliser; °, large particle (12 μm) infection generated by the FFAG. Mean values are indicated by the solid and dotted lines for the small particle and large particle infections respectively (*n* = 5). Significant differences between the tissue loads are indicated by ^*^(*p* < 0.05) and ^**^(*p* < 0.01), respectively.

**Figure 3:**
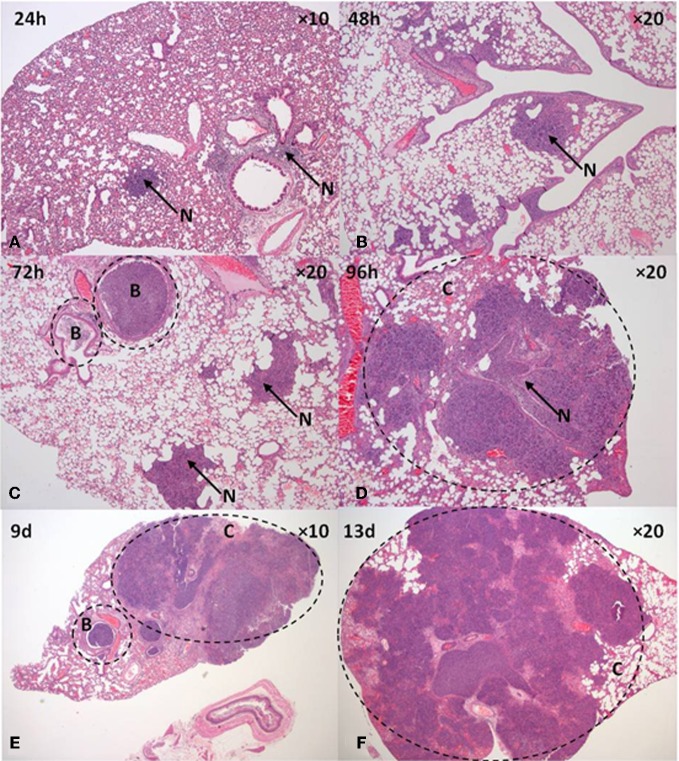
**Pathology of the lung after inhalation of *B. pseudomallei* K96243 aerosolized in 12 μm aerosol particles. (A)** 24 h, **(B)** 48 h, **(C)** 72 h, **(D)** 96 h, **(E)** 9 d, and **(F)** 13 d. B, bronchiole, C, consolidated tissue, N, suppurative inflammation.

The temporal progression of inhalational melioidosis caused by deposition of *B. pseudomallei* within either the LRT or URT was characterized by bacteriological and histopathological analysis; summarized in Table 1. Inhalation of the aerosol produced by the Collison nebuliser (1 μm particles) resulted in the deposition of 2.93 ± 0.3 × 10^2^ and 1.6 ± 0.1 × 10^1^ cfu in the lungs and nasal passages, respectively. Conversely, the inhalation of the aerosol produced by the FFAG (12 μm particles) resulted in the deposition of 5.2 ± 4.1 × 10^1^ and 3.0 ± 0.2 × 10^2^ cfu in the lungs and nasal passages, respectively. Irrespective of the aerosol generator used, the resultant infection was characterized by primary pneumonic infection of the lung. Significant differences were observed in the bacterial load in the lungs at 0 and 24 h post-challenge (Figure 2; *p* = 0.034), however, by 96 h post-challenge the loads were similar irrespective of the size of the particles inhaled reaching 1.3–1.5 × 10^9^ cfu g^−1^ (*p* = 0.96). Initially at 24 h post-challenge small inflammatory foci were observed with neutrophilic exudates into the alveoli and necrosis indicative of acute alveolitis that increased in size as the infection developed (Figure 3). The foci were present throughout the cranial, middle and caudal lobes. Inflammatory exudates were observed occupying bronchiolar airways by 72 h post-challenge (Figure 3C). In the majority of mice that inhaled both 1 μm (100%; 8/8) or 12 μm particles (82%; 14/17) the inflammatory foci merged into large areas of consolidation comprising predominantly neutrophils and fewer macrophages, bacterial colonies, and necrotic tissue (Figure 3D). Most mice succumbed to infection by 72–120 h, however, some survived for prolonged periods of up to 21 d post-challenge. Interestingly at this stage, the lungs were almost completely consolidated (Figures 3E,F), however, surprisingly there were no obvious signs of severe respiratory distress other than the mice demonstrating clinical signs of ruffled fur and a hunched posture.

**Table 1:** **Comparative histopathological and bacteriological progression of inhalational melioidosis infections in the major tissues affected**.

**Particle size**	**Tissue^*a*^**	**Progression of infection post-challenge (h)^*b*^**				
		24	48	72	96	120+^*c*^
Small particle aerosol (1–3 μm)	Nasal mucosa	−	−	−	+	N.D
	NALT	−	−	−	−	N.D
	Cervical node	−	+	+	+	N.D
	Mediastinal node	−	+	++	+++	N.D
	Lung	−	++	++++	+++++	N.D
	Pharynx/Trachea	−	+	+	++	N.D
	Olfactory epithelium	−	+	+	++	N.D
	Olfactory nerves/tracts	−	+	+	++	N.D
	Olfactory bulb	−	−	−	+	N.D
	Brain	−	−	−	−	N.D
	Spleen	−	+	++	+++	N.D
	Liver	−	+	++	+++	N.D
	Thymus	−	+	+	++	N.D
	Blood	−	+	++	++++	N.D
Large particle aerosol (12 μm)	Nasal mucosa	+	++	++	+++	+++++
	NALT	−	+	++	++	++
	Cervical node	+	++	+++	+++++	+++++
	Mediastinal node	−	−	+	++	++
	Lung	−	++	+++	++++	++++
	Pharynx/Trachea	−	+	++	+++	+++
	Olfactory epithelium	++	++++	+++++	+++++	+++++
	Olfactory nerves/tracts	−	++	+++	+++++	+++++
	Olfactory bulb	−	−	++	++++	+++++
	Brain	−	−	−	++	++++
	Spleen	−	++	+++	+++	++++
	Liver	−	++	+++	+++	++++
	Thymus	−	+	++	+++	+++
	Blood	−	+	+++	++++	N.D

ND, not determined.

a Intestine, stomach, Peyer's patches, mesenteric lymph nodes, bone marrow, and oesophagus were negative for pathology until 96 h post-infection in both 1 and 12 μm inhalational infections when bacteria were observed in the Peyer's patches and mesenteric lymph nodes correlating with bacteraemia.

b Severity of pathological changes and bacteriological load is scored subjectively according to the methods: −, none; +, mild; ++, moderate; +++, marked; ++++, major; +++++, severe.

c Some mice developed chronic infection and survived for up to 20 d post-challenge.

**Figure 4:**
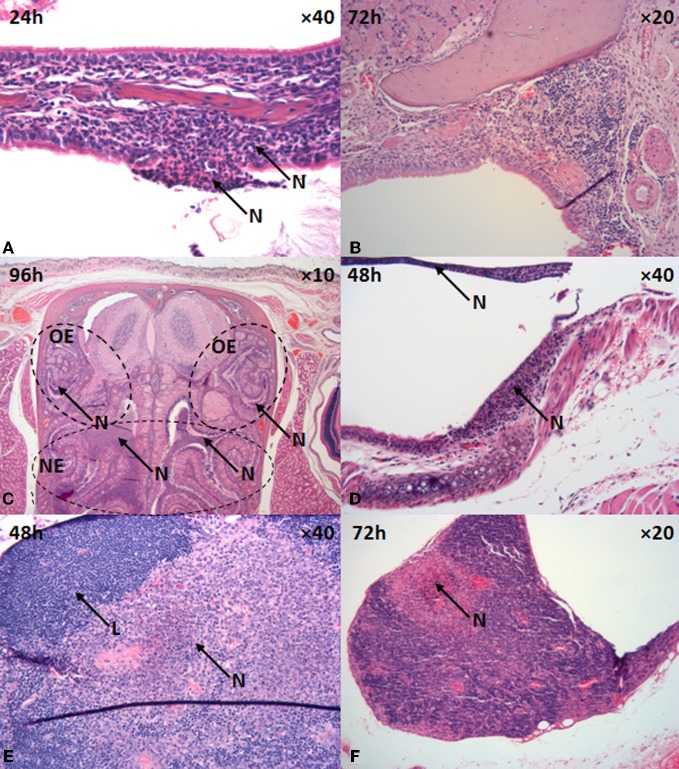
**Upper respiratory tract pathology in mice that inhaled 12 μm aerosol particles containing *B. pseudomallei* K96243. (A)** nasal epithelium, **(B)** NALT, **(C)** nasal respiratory epithelium, **(D)** trachea, **(E,F)** cervical lymph nodes. L, lymphocyte, N, suppurative inflammation, NE, nasal epithelium, OE, olfactory epithelium.

However, differences were observed in the degree of URT and neurological involvement between the infections. Differences in the bacterial load within the nasal cavity were observed over the time-course of experimentation with significance at 24 h post-challenge (*p* = 0.009; Figure 2). Bacterial loads in the nasal cavity increased reaching 1.3 ± 0.5 × 10^4^ and 7.1 ± 4.1 × 10^5^ cfu ml^−1^ at 72 h post-challenge in mice that inhaled 1 and 12 μm particles, respectively (*p* = 0.073). By 48 h post-challenge, significant pathological changes of epithelium of the nasal mucosa was observed in mice exposed to *B. pseudomallei* with 12 μm aerosol particles. The olfactory epithelium was preferentially targeted with 82% (14/17) of the mice demonstrating infection in this region compared to the respiratory mucosa of the nasal cavity (71%; 12/17). As early as 24 h post-challenge, neutrophil infiltration of the lamina propria and exudation in the nasal passages with associated bacteria can be observed (Figure 4A).

NALT was preferentially infected in mice that inhaled *B. pseudomallei* within 12 μm particles (41%; 7/17) compared to 1 μm particles (12.5%; 1/8) with lymphocytic degeneration and necrosis, neutrophilic infiltration and large numbers of bacteria present within the necrotic tissue (Figure 4B). Interestingly, those mice that survived past 120 h infection and subjected to histological analysis did not show evidence of NALT involvement (0%; 0/3). Irrespective of the size of the particles inhaled, the pharynx and trachea were infected similarly at 75% (6/8) and 80% (12/15) for mice that inhaled *B. pseudomallei* within 1 and 12 μm particle aerosols, respectively. In the pharynx, exudation into the luminal space of sloughed epithelial cells was observed in 6–12% of mice. An ulcerative and suppurative tracheitis occurred in 75–80% of mice with sloughing of epithelial cells across extensive areas, neutrophil infiltration, and exudation into the tracheal lumen (Figure 4D). The severity of pharyngeal and tracheal pathology appeared earlier and was more extensive in mice exposed to the 12 μm particle aerosol (Table 1). In mice that inhaled 12 μm particles, the cervical lymph nodes became infected in 71% of mice (12/17) by 24 h post-challenge. Pathological changes in the cervical lymph nodes included multifocal necrosis, neutrophilic infiltration, and granulomatic encapsulation by macrophages as the early stages of abscessation (Figures 4E,F). In contrast, only 38% of mice (3/8) that inhaled *B. pseudomallei* within 1 μm particles displayed similar histological changes in the cervical lymph nodes.

**Figure 5:**
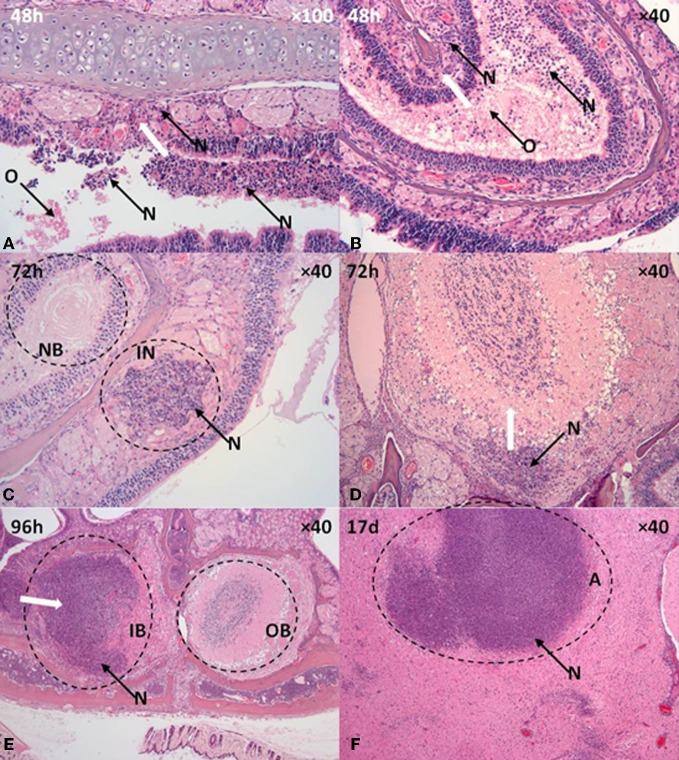
**Olfactory and neurological pathology in mice that inhaled *B. pseudomallei* K96243 in 12 μm aerosol particles. (A,B)** Olfactory epithelium, **(C,D)** Olfactory nerves, **(E)** Olfactory bulb, **(F)** brain. A, abscess, IB, infected olfactory bulb, IN, infected neurone, N, suppurative inflammation, OB, olfactory bulb, NB, neuronal bundle. White arrows indicates direction of neutrophil taxis and inflammation.

In mice that inhaled 12 μm particles, the inflammatory response in the nasal cavity progressed over time, with extensive neutrophilic infiltration and bacterial proliferation encompassing both the respiratory and olfactory epithelia observed at 96 h post-challenge (Figures 4C and 5A,B). Neutrophilic infiltration, bacterial infection, and inflammation of the olfactory nerves/tract (80%; 12/15) and olfactory bulb (60%; 9/15) were observed by 48–96 h post-challenge. Histological analysis indicated neutrophilic infiltration into the axonal bundle of the olfactory neurons followed by temporal passage into the olfactory bulb (Figures 5C–E). Generally the infection was acute enough for the mice to be humanely culled by 96–120 h post-challenge, however, some mice survived for prolonged periods up to 20 d post-challenge developing a chronic infection. In 33% of mice (2/6) that developed chronic infection (2/6) histological analysis revealed infection of the olfactory tracts extending through the ethmoidal foramina into the olfactory bulb of the brain. Microabscesses were also observed in the medulla and brainstem with the larger abscesses (~0.5 mm) located in the ventrorostral and mid-regions of the brain (caudate putamen, olfactory areas) (Figure 5F). Areas of suppurative encephalitis can be observed with neutrophilic infiltration into the brain tissue. Infection of the olfactory and respiratory epithelium was observed in mice that inhaled 1 μm particles containing *B. pseudomallei*, however, comparatively this occurred later during the infection by 96 h and to a lesser degree (37%; 3/8). Furthermore, only 12% of mice (1/8) that inhaled 1 μm particles demonstrated pathology of the olfactory bulb.

Bacteraemia was observed at similar levels at 48 h post-challenge in both inhalational infections, 0.57–1.4 × 10^2^ cfu ml^−1^ (*p* = 0.066) increasing to 1.3–2.2 × 10^5^ cfu ml^−1^ by 96 h post-challenge (*p* = 0.19; Figure 2). Bacteraemic and/or lymphatic spread to visceral organs such as the spleen and liver was evident with hyperplasia in the red pulp, and multifocal microabscess formation observed in both tissues as early as 48 h post-challenge contributing at later time-points to spleno- and hepatomegaly. In the spleen, the microabscesses merged into larger abscesses at the later time-points (>96 h). The bacterial loads in the spleen and liver were highly variable, particularly at 96 h post-challenge, probably due to the degree of abscessation observed (Figure 2). For example at 96 h post-challenge, although the spleen bacterial loads in the mice exposed to the 1 μm particle aerosol was 5.85 ± 5.72 × 10^6^ cfu g^−1^ and greater than that observed in mice exposed to 12 μm particle aerosols (4.79 ± 7.37 × 10^5^ cfu g^−1^), this was not a significant difference due to the variance in loads observed between individual mice (*p* = 0.46). The thymus was infected similarly in both the infections caused by the inhalation of 1 and 12 μm particle aerosols at 50% (4/8) and 53% (9/17), respectively, with lymphocytic depletion and increased tangible body macrophages (Table 1). Pathology in the inguinal lymph nodes was only observed at 96 h post-challenge in 12% (2/17) of mice that inhaled 12 μm particle aerosols. The mesenteric lymph nodes only displayed pathological changes late in the infection in mice that inhaled 12 μm particles (29%; 5/17) displaying distension of the medulla and/or subcapsular sinuses and marked medullary histiocytosis, probably indicative of bacteraemic spread. No other gastrointestinal pathology was observed irrespective of the size of particles inhaled.

### Prophylactic intervention with co-trimoxazole affects survival in the large particle inhalational infection model

**Figure 6:**
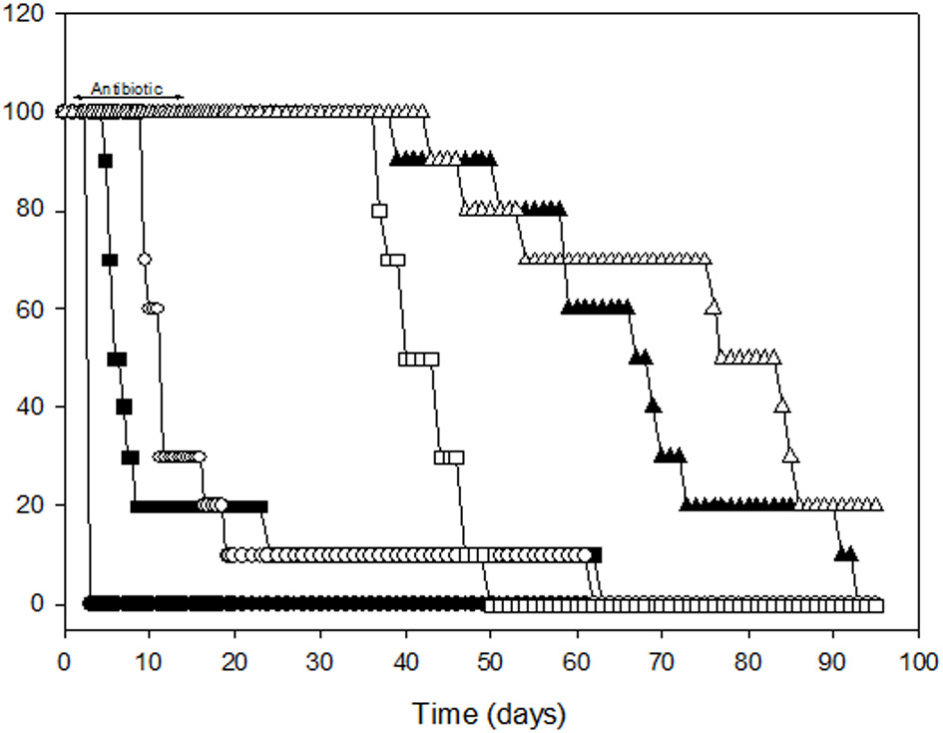
**Efficacy of antimicrobial chemotherapy with doxycycline or co-trimoxazole against infection caused by the deposition of *B. pseudomallei* K96243 within the lower or upper respiratory tract in 1 (closed symbols) or 12 μm (open symbols) aerosol particles, respectively.** Circles, untreated control mice; squares, doxycycline treated mice; triangles, co-trimoxazole treated mice.

Little published data exist on the *in vivo* efficacy of antimicrobials against experimental melioidosis, particularly via inhalational challenge. In human cases of melioidosis, prolonged combinatorial antimicrobial treatment is required with ceftazidime, in conjunction with imipenem, co-trimoxazole, amoxicillin-clavulanate, doxycycline, or chloramphenicol. However, doxycycline (100 mg twice daily) or co-trimoxazole (960 mg twice daily) are the prophylactic antibiotics of choice in a mass-casualty setting resulting from an intentional release of *B. pseudomallei* and hence the focus for this study (Chaowagul et al., 2005; Gilad et al., 2007; HPA, 2008). A comparison was made between the efficacy of doxycycline and co-trimoxazole to protect mice against *B. pseudomallei* deposited in the respiratory tract within either 1 or 12 μm particle aerosols (Figure 6). The MICs of doxycycline and co-trimoxazole (trimethoprim/sulfamethoxazole) for *B. pseudomallei* K96243 are 0.5 and 32/16 μg ml^−1^, respectively (Barnes, pers. comm.). Mice were challenged with approximately 100 MLDs of *B. pseudomallei* within either 1 or 12 μm particle aerosols. The actual retained doses were 5.7 ± 1.1 × 10^2^ cfu and 8.7 ± 0.9 × 10^2^ cfu for the 1 or 12 μm particle aerosols within the lungs and nasal passages, respectively. Irrespective of particle size, all of the untreated control mice succumbed to the challenge. The MTD for the untreated control mice were significantly different at 2.5 ± 0 and 16.0 ± 5.2 d for the 1 and 12 μm particle aerosol infections, respectively (*p* = 0.028).

Antimicrobial therapy was commenced, with a once or twice-daily regimen of doxycycline (50 mg kg^−1^) or co-trimoxazole (240 mg kg^−1^) at 12 h intervals for 14 d. Doxycycline was significantly more effective against the 12 μm particle infection compared to the 1 μm particle infection. Despite doxycycline treatment, 90% of mice that inhaled 1 μm particles succumbed by 26 d post-infection. Survival was increased to 69 d post-infection in doxycycline treated mice that inhaled *B. pseudomallei* within 12 μm particle aerosols. This was exemplified by significantly different MTDs for the 1 and 12 μm particle infections at 13.0 ± 5.1 and 61.4 ± 1.5 d, respectively (*p* = 0.0001). Administration of co-trimoxazole significantly prolonged survival with all of the animals surviving until day 58 irrespective of the particle size inhaled; however, clearance was not observed and the mice started developing signs of infection 2–3 weeks after antimicrobial dosing was completed and began succumbing to infection 6–7 weeks post-infection. The experiment was terminated 93 d post-infection with 0% and 20% survival observed in groups that inhaled *B. pseudomallei* within 1 and 12 μm particles respectively. The MTDs for the 1 and 12 μm particle infections were 68.6 ± 4.5 and 88.0 ± 6.4 d, respectively (*p* = 0.0005). In all of the antibiotic treated groups, bacteriological analysis of the lungs and spleens indicated that clearance had not occurred. Bacterial burdens in the lungs were significantly increased in mice treated with doxycycline (1.1 ± 0.11 × 10^8^ cfu g^−1^) compared to co-trimoxazole treated mice (5.8 ± 2.4 × 10^4^ cfu g^−1^; *p* = 0.007). No difference was observed in bacterial loads from spleens irrespective of the antibiotic treatment received with numbers ranging from 1.1 to 8.1 × 10^7^ cfu g^−1^ (*p* = 0.14). Post-mortem analysis of mice in groups that succumbed to infection 6–7 weeks post-treatment indicated large abscesses present within the lungs, spleens and often the cervical lymph nodes.

## Discussion

Increased incidence of inhalational melioidosis within endemic countries in Southeast Asia and Northern Australia is associated with the monsoon season (Currie and Jacups, 2003; Cheng et al., 2006, 2008). Exposure to airborne *B. pseudomallei* would be predicted to increase in prevalence during the rains as bacteria migrate through the soil, collect in puddles and are aerosolized due to winds or human/animal activity into the humid atmosphere. Clustered human and animal outbreaks of inhalational melioidosis have been reported to be associated with heavy rainfall, localized flooding, gusting winds, and contamination of an aerator unit in a water treatment plant (Ketterer et al., 1986; Inglis et al., 1999, 2000). However, the particle size distribution of the inhaled aerosols was unknown and a clear link between particle size and pathology has not been established in such incidences.

The data reported in this study indicates that aerosol particle size can affect the infectivity and pathogenesis of *B. pseudomallei* in the murine model. Despite clear differences in deposition profiles between the LRT and URT, the infections produced by the inhalation of *B. pseudomallei* within 1 and 12 μm particle aerosols had similarities particularly with respect to the temporal apparition and progression of pulmonary pathology. This can be attributed to the fact that although predominantly comprising larger particles, the aerosol generated by the FFAG, will also comprise some 1–3 μm particles that would be predicted to deposit in the lungs (Thomas et al., 2008). The low MLD of 4 cfu in the Balb/c murine model of *B. pseudomallei* infection caused by lung deposited 1 μm particles means that it is impossible to generate purely LRT or URT infections on the basis of particle size simply due to the dynamics of aerosol production and the kinetics of aerosol deposition. However, this lack of a defined aerosol generated by the FFAG is probably a more realistic reflection of an environmental aerosol that will be naturally polydisperse. The MLD of 4 cfu for *B. pseudomallei* K96243 contained within 1 μm particles is similar to the 5–20 cfu for *B. pseudomallei* strains 1026b, BRI NCTC 4845, 576, and KHW in the Balb/c murine model (Jeddeloh et al., 2003; Tan et al., 2008; Titball et al., 2008; Lever et al., 2009). Bacterial genetic background can influence MLD as evidenced by the MLDs of 27 and 423 cfu reported for *B. pseudomallei* strains 1026b and KHW in the C57BL/6 murine strain (Jeddeloh et al., 2003; Tan et al., 2008). In addition the temporal kinetics of infection for the 1 μm particle inhalational infection are similar to reports in the literature for various animal models (murine, marmoset) with initial lung infection followed by dissemination to visceral tissues once bacteraemia occurs at approximately 24 h post-infection (Jeddeloh et al., 2003; Lever et al., 2009; Nelson et al., 2011).

The MLD of 12 cfu obtained in this study with *B. pseudomallei* within 12 μm particles is three-fold greater than that obtained in mice that inhaled 1 μm particles. Furthermore, the MTD was significantly increased when the cumulative data from all experiments was examined. MTDs of 73.8 ± 11.3 h and 174.7 ± 14.9 h were obtained for mice that inhaled 1-5 MLDs of *B. pseudomallei* within 1 and 12 μm particle aerosols respectively (*n* = 25, *p* = 0.007). Differences were observed as a result of deposition within the URT associated with increased pathology in the nasal mucosa and associated lymphoid tissue and neural structures. Extensive ulceration of the nasal epithelium was observed with preferential localization on the olfactory epithelium. An acute suppurative neuritis ensued as bacteria and inflammatory cells moved up the olfactory neuronal bundles into the olfactory bulb via the ethmoidal foramina. The anterior and posterior ethmoidal foramina are small gaps in the cribriform plate of the skull through which the innervating neurons (and blood vessels) of the nasal cavity directly pass into the brain. This neurological path to the brain was independent of bacteraemic spread as the olfactory nerves were demonstrating significant pathology at 24–48 h post-challenge when bacteraemia was minimal. Interestingly, these pathological changes and bacterial colonization has been observed after intranasal administration of *B. pseudomallei* to Balb/c mice (Owen et al., 2009). In this study, collation of all the murine data indicated that paralysis was observed in 10/100 (10%) and 54/130 (42%) mice that succumbed to infection after inhaling *B. pseudomallei* within 1 and 12 μm particles, respectively (*p* = 0.0004). Paralysis is an indicator of neurological dysfunction and this data highlight the propensity of *B. pseudomallei* to infect neurological tissues and that infection via deposition in the URT is a predisposing factor.

*B. pseudomallei* can attach to human respiratory epithelial cell lines through via attachment to gangliosides such as asialo-GM1 and asialo-GM2 (Ahmed et al., 1999; Gori et al., 1999). Indeed, attachment can be inhibited by incubation in the presence of the disaccharide moieties (GalNAcβ1-4Gal and GalNAcβ1-3Gal) present within the ganglioside structure (Thomas and Brooks, 2004). The prevalence of these gangliosides increase on the apical surface of damaged and repairing airway epithelial cells (de Bentzmann et al., 1996). Increased damage to the nasal, olfactory, pharyngeal, and tracheobronchial epithelia was observed over the duration of the infection and increased expression of binding ligands on the damaged cells may account for the prevalence of *B. pseudomallei* within these locations. Gangliosides, including asialo-GM1 and asialo-GM2 are also prevalent on the cell surface of neurones (Yu et al., 2011). The preference of *B. pseudomallei* for the neurological pathway after deposition in the URT may be due to its ability to bind to gangliosides such as asialo-GM1 and asialo-GM2, and perhaps others as yet unidentified.

Direct infection of the brain via retrograde axonal transport through the olfactory neurone from the nasal cavity has been observed with viruses, bacteria, and protozoans including Japanese encephalitis virus, Eastern equine encephalitis virus, Venezuelan equine encephalitis virus, Influenza virus, Equine herpesvirus-9, *Streptococcus pneumoniae*, *Neisseria meningitidis*, *Balamuthia mandrillaris* and *Naegleria fowleri* (Danes et al., 1973a,b,c; Martinez, 1977; Jarolim et al., 2000; van Ginkel et al., 2003; Kiderlen and Laube, 2004; Roy et al., 2009; Yamada et al., 2009; El-Habashi et al., 2010; Sjölinder and Jonsson, 2010; Steele and Twenhafel, 2011; Schrauwen et al., 2012). Even prions have been demonstrated to shed into the nasal cavity from the brain using the reverse of this transport process (Corona et al., 2009; Bessen et al., 2010). However, it is uncertain whether the olfactory pathway as a route of infection extrapolates to NHPs and humans. Indeed, the rodent olfactory epithelium has a much larger surface area in comparison to primates (Harkema, 1991; Philström et al., 2005). This perhaps predisposes rodents to infection via this route.

Neurological melioidosis is an infrequent complication of human infection presenting as encephalomyelitis in the presence or absence of multifocal abscessation (Lee and Chua, 1986; Pelekanos and Appleton, 1988; Kasantikul et al., 1992; Woods et al., 1992; Padiglione et al., 1998; Chadwick et al., 2002; Koszyca et al., 2004). It is assumed that infection of the brain is a result of bacteraemic spread; however, the olfactory neuronal pathway has not been examined as a potential mechanism of infection via the URT in primates. Interestingly, an association between sinusitis in the frontal or maxillary sinuses of the URT and five cases of cerebral melioidosis has been observed with the authors highlighting that the sinuses “may represent a source of entry into the CNS” (Chadwick et al., 2002). One NHP study has been published on *B. pseudomallei* infection in the marmoset resulting from the inhalation of a small particle aerosol where no brain histology was observed (Nelson et al., 2011). This is expected because the 1–3 μm particle aerosols generated deposit in the alveolar region of the lung in the marmoset; however, it does not preclude involvement of the olfactory neuronal pathway and brain if deposition predominantly occurred within the nasal cavity.

Increased involvement of the URT lymphoid tissues (NALT, cervical lymph nodes) were observed when *B. pseudomallei* were inhaled within 12 μm particles. This is similar to other pathogens infective via the URT, including *Yersinia pestis* and *Bacillus anthracis* (Thomas et al., 2009, 2010). URT pathology resulting from *B. pseudomallei* infection has been observed in US soldiers returning from Vietnam and patients in South-East Asia presenting amongst other symptoms with severe pharyngitis with tonsillar exudates, suppurative cervical lymphadenopathy, and acute pansinusitis (Patterson et al., 1967; Lim et al., 2001). This pathology is consistent with the murine infection produced after the inhalation of 12 μm aerosol particles. Despite significant clearance to the gastrointestinal tract, pathology was not observed in infections caused by the inhalation of 1 or 12 μm particles. Infection via the gastrointestinal tract can occur in the murine model, but only with high doses in the region of 10^8^ cfu (West et al., 2010). These doses far exceed the approximate 10^4^ cfu cleared to the stomach in this study.

Co-trimoxazole has previously been demonstrated to have higher levels of efficacy (100%) compared to doxycycline (80%) in the small particle inhalational murine model provided treatment commenced prior to 48 h post-infection (Sivalingam et al., 2008). In this study, irrespective of the site of deposition, inhalational melioidosis was difficult to treat (Figure 6). One difference may be the oral delivery method used for antibiotic dosing, Sivalingam et al. (2008) used oral gavage and given the viscous nature of the co-trimoxazole preparation (Septrin®) in particular, it is difficult to ascertain whether the mice swallowed the entire dose via oral pipetting and at times a noticeable quantity remained around the jowls; furthermore this study was conducted for a greater period than 21 d post-infection to capture relapse. Similar to the results of Sivalingam et al. (2008), Co-trimoxazole was a significantly better treatment than doxycycline for inhalational melioidosis. This is possibly due to the fact that co-trimoxazole is bactericidal against *B. pseudomallei* whilst doxycycline is bacteristatic as evidenced during *in vitro* time-kill assays (Barnes pers. comm.). Hence, the potential to reduce bacterial burden within extracellular locations prior to *B. pseudomallei* reaching areas where it may lie latent is greater with co-trimoxazole. However, it must be noted that complete efficacy was not observed in this study as relapse occurred once treatment was terminated highlighting the intrinsic resistance of this bacterium to antimicrobial therapy. This is probably due to a number of reasons including bacterial resistance factors (i.e., efflux pumps) and lack of understanding mechanisms of latency and pathological location (i.e., intracellular niche, abscessation, neurological tissues). Further research to investigate aspects such as latency and optimization of treatment regimes are currently underway.

In addition to the respiratory route of infections, the olfactory nervous system may warrant attention as a novel target for disease prevention in *B. pseudomallei*. Certainly, little is known in this region regarding host physical and immune strategies in preventing infection (Kalinke et al., 2011). It is interesting to speculate whether the difficulties in treating inhalational infections within animal models are a reflection of the multiple portals of entry a pathogen may use to invade the body. Furthermore, intranasal instillation may not be the most appropriate method of evaluating vaccine candidates and/or antimicrobial therapies for treatment of respiratory infections due to the propensity to target pathogens to not only the LRT, but also the URT (and indeed the GI tract) with the relative concentrations at each site often undetermined. This study indicates that in addition to inhalational melioidosis initiating in the lungs, other sites of the respiratory tract should be considered as portals of entry and that these may present further complications to effective therapy.

### Conflict of interest statement

The authors declare that the research was conducted in the absence of any commercial or financial relationships that could be construed as a potential conflict of interest.
